# Comparison of two metaphyseal-fitting (short) femoral stems in primary total hip arthroplasty: study protocol for a prospective randomized clinical trial with additional biomechanical testing and finite element analysis

**DOI:** 10.1186/s13063-019-3445-x

**Published:** 2019-06-17

**Authors:** I. Tatani, A. Panagopoulos, I. Diamantakos, G. Sakellaropoulos, Sp Pantelakis, P. Megas

**Affiliations:** 1 Orthopaedic Department, University Hospital of Patras, Patras, Greece; 2 Laboratory of Technology and Strength of Materials, Department of Mechanical Engineering and Aeronautics, University of Patras, Patras, Greece; 3 Department of Medical Physics, School of Medicine, University of Patras, Patras, Greece

**Keywords:** Total hip arthroplasty, Short stem, Biomechanical testing, Finite element analysis, Clinical outcome

## Abstract

**Background:**

Total hip replacement has recently followed a progressive evolution towards principles of bone- and soft-tissue-sparing surgery. Regarding femoral implants, different stem designs have been developed as an alternative to conventional stems, and there is a renewed interest towards short versions of uncemented femoral implants. Based on both experimental testing and finite element modeling, the proposed study has been designed to compare the biomechanical properties and clinical performance of the newly introduced short-stem Minima S, for which clinical data are lacking with an older generation stem, the Trilock Bone Preservation Stem with an established performance record in short to midterm follow-up.

**Methods/design:**

In the experimental study, the transmission of forces as measured by cortical surface-strain distribution in the proximal femur will be evaluated using digital image correlation (DIC), first on the non-implanted femur and then on the implanted stems. Finite element parametric models of the bone, the stem and their interface will be also developed. Finite element predictions of surface strains in implanted composite femurs, after being validated against biomechanical testing measurements, will be used to assist the comparison of the stems by deriving important data on the developed stress and strain fields, which cannot be measured through biomechanical testing. Finally, a prospective randomized comparative clinical study between these two stems will be also conducted to determine (1) their clinical performance up to 2 years’ follow-up using clinical scores and gait analysis (2) stem fixation and remodeling using a detailed radiographic analysis and (3) incidence and types of complications.

**Discussion:**

Our study would be the first that compares not only the clinical and radiological outcome but also the biomechanical properties of two differently designed femoral implants that are theoretically classified in the same main category of cervico-metaphyseal-diaphyseal short stems. We can hypothesize that even these subtle variations in geometric design between these two stems may create different loading characteristics and thus dissimilar biomechanical behaviors, which in turn could have an influence to their clinical performance.

**Trial registration:**

International Standard Randomized Controlled Trial Number, ID: ISRCTN10096716. Retrospectively registered on May 8 2018.

**Electronic supplementary material:**

The online version of this article (10.1186/s13063-019-3445-x) contains supplementary material, which is available to authorized users.

## Background

Total hip arthroplasty (THA) for the treatment of advanced hip osteoarthritis is considered one of the most successful surgical procedures of the last century, aiming to relieve pain and improve function of the hip joint [1–5]. Conventional, cementless long stems have shown 10-year survival rates of more than 90% [6–8] but as THA is increasingly performed in younger and more active patients [9], recent implant developments are aimed towards minimizing tissue damage and preserving bone stock without compromising implant stability [10]. As the novel concept of patient fast-tracking with less invasive interventions concerns not only the surgical approaches or the blood loss but the materials as well, many manufacturers have designed short-stem implants using different proximal geometries and design philosophies. Short stems have been designed in a way to better fit in the metaphysis of the proximal femur thus reproducing a biomechanical behavior more similar to the physiological bone. The clinical impact of these innovations would be a more accelerated rehabilitation program, improved long-lasting functional outcome and mainly, preservation of bone stock for future revisions. A rigid primary fixation and a larger area of metaphyseal contact are the essential requirements for a successful osteointegration of these shorter stems with an ultimate goal to produce a more physiological load transfer to the femur thus eliminating the stress-shielding effect of the standard long stems. However, these hypothetical benefits still need to be verified [11].

The idea of short-stem hip arthroplasty was first reported by Judet and Judet in 1940 [12] with less satisfactory outcomes [13, 14]. The next model, the Mayo Conservative Hip (Zimmer, Warsaw, IN, USA) was designed to anchor in a larger fixation area [15] and has been in clinical practice for more than 20 years since. In 2000, Morrey et al. [16] reported a 94% survival rate after a mean follow-up period of 6.2 years. Since then, a wide variety of new models of short stems have emerged, with differences in operative technique and method of fixation [17, 18]. Due to a lack of adequate regulation, many innovative hip implants were easily adopted by clinicians without sound premarketing testing and high-quality evidence supporting their clinical use.

From the biomechanical perspective, initial stability and initial lack of motion at the bone-prosthesis interface are essential for long-term survival rates and, therefore, accuracy of host bone preparation and prosthesis design are crucial, whereas implant surface texture and quality of the bone-implant contact determine secondary stabilization of the prosthesis. Various techniques of measuring the cortical deformation pattern as a result of implantation with different prostheses have been described in the literature. Experimental testing employing strain gauges constitutes the most common technique for measuring strain fields in the assessment of post-implantation bone response in vitro [19–23]. However, a major limitation of this technique is that the information is provided from the attachment site only. Recently, digital image correlation (DIC), a non-contact optical metrology method, has gained popularity due to its capacity to extract full-field surface-strain measurements of objects subjected to external loads [24–27]. In addition, the Finite element analysis (FEA) method has been well established as another tool of calculating deformation, strain and stress patterns in implanted bones [28, 29]. Finite element (FE) models provide three-dimensional (3D) strain predictions in implant-bone constructs and enable analyses of the developed stress and strain fields in areas where strains cannot be experimentally measured, such as the bone-implant interface. However, the assumption of simplified bone material properties, geometry and loading conditions is often made in most of parametric 3D FE models and thus their validation using experimental tests is recommended wherever it is possible [24, 30, 31].

**Fig. 1:**
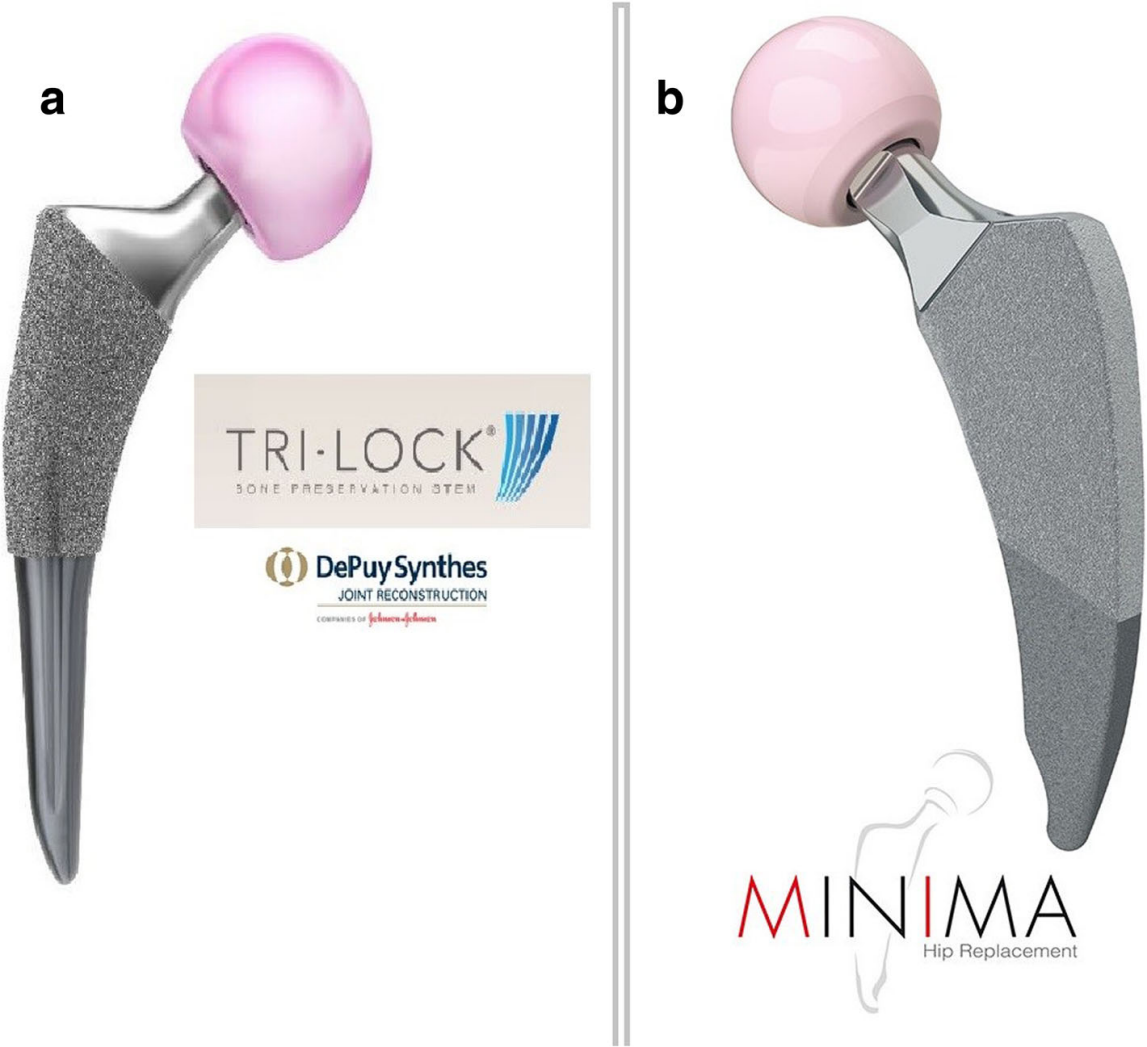
The two different stems that will be compared in the study **a**. Trilock Bone Preservation Stem, **b**. Minima S

The proposed study has been designed to compare the biomechanical properties, based on both biomechanical tests and FE models, as well as the clinical performance of two different metaphyseal-fitting short stems; the TRI-LOCK Bone Preservation Stem (DePuy Orthopaedics Inc. Warsaw, IN, USA) and the Minima S Femoral Stem (Lima corporate Villanova di San Daniele, Italy) (Fig. 1).

In the experimental study, the transmission of forces as measured by cortical surface-strain distribution in the proximal femur will be evaluated using DIC, first on the non-implanted femur and then on the implanted TRI-LOCK Bone Preservation Stem (BPS) and Minima S femoral stems. The strain patterns of the non-implanted femur will served as the control group. The primary outcome would be to check the presence of adequate metaphyseal anchorage and the effect of the two different-design short stems on the strain patterns compared to the unimplanted femur.

Finite element parametric models of the bone, the stem and their interface will be also developed. Finite element predictions of surface strains in intact and implanted composite femurs, after being validated against biomechanical testing measurements, will be used to assist the comparison of the stems by deriving important data on the developed stress and strain fields, which cannot be measured through biomechanical testing.

A prospective randomized comparative clinical study between these two types of short stems, will be also conducted to determine (1) their clinical performance up to 2 years’ follow-up using validated scoring instruments and gait analysis parameters; (2) stem fixation and bone remodeling using a detailed radiographic analysis and (3) incidence and types of complications if any.

## Methods/design

### Biomechanical study

The experimental work will be undertaken in the Laboratory of Technology and Strength of Materials at the Department of Mechanical Engineering and Aeronautics of Patras University.

#### Implant systems

Two implant systems will be used, the TRI-LOCK Bone Preservation Stem (DePuy Orthopaedics Inc. Warsaw, IN, USA) and the Minima S Femoral Stem (Lima corporate Villanova di San Daniele, Italy). The technical characteristics of these stems are summarized below:

- Tri-Lock BPS (DePuy, Johnson & Johnson, Warsaw, IN, USA) is a short, tapered-wedge, proximally porous-coated titanium femoral stem. Compared to its clinically successful predecessor, the Tri-Lock standard stem, the BPS stem is shorter, has a narrower distal segment, and features a curved distal tip. Also, the Tri-Lock BPS has a highly porous pure titanium (“GRIPTION®”) coating on the proximal 50% portion that is engineered to provide an increased surface roughness when compared to POROCOAT® porous coating, which is on the original TRI-LOCK stem. The TRI-LOCK BPS is available in 13 stem sizes (size 0–12/length 95–119 mm) with standard and high offset options for all stem sizes. The high offset option provides direct lateralization, thus increasing offset without affecting either the leg length or the neck-shaft angle, which remains constant at 130^0^
- Minima S Monolithic Femoral Stem (Lima corporate Villanova di San Daniele, Italy) is a short, curved, four-tapered, proximally porous-coated titanium femoral stem. It is comprised of 12 stem sizes (size 1–12/length 82–118 mm) in standard and lateralized configuration. The standard versions have a neck-shaft angle of 134°, while the lateralizing versions have a neck-shaft angle of 131°. The Minima S stem has a tapered and medially side-sharpened tip aiming to reduce the risk of contact with the cortical medial wall

#### Study aims

Although both stems belong to the same short-stem family, the Tri-Lock BPS is a stem with a geometrical design similar to conventional long stems (its design is based on its clinically successful predecessor, the Tri-Lock standard stem), but shorter with a narrower distal segment. On the other hand, the Minima S stem is even shorter, has an anatomic shape following the natural curvature of the medial calcar, preventing breach of the greater trochanter and a medially sidecut tip to reduce the risk of contact with the cortical medial wall.

We can hypothesize that the above variations, regarding stem length and geometric design, may create differences in strain distribution and thus dissimilar biomechanical behaviors. Specifically, we sought to determine if the implantation of a shorter, anatomically shaped stem would produce a pattern of strain distribution closest to normal, reduce the risk of distal locking and proximal offloading. Therefore, strain patterns after implantation of the Minima S stem are compared to both strain patterns without an implanted stem and strain patterns following implantation of the Trilock BPS stem.

#### Preparation of the femurs

Seven fourth generation, medium composite, femurs from Sawbones Europe (Malmö, Sweden) with identical design and material properties will be used in accordance to previous biomechanical studies [13, 32–37]. Three different composite femoral bones for each prosthesis will be prepared by the same investigator (I.T.) according to the manufacture’s surgical technique. Also, a set of three tests will be repeated for each specimen. The quality of implantation in terms of correct implant size and positioning (anteversion) will be assessed using antero-posterior and lateral radiographs. After the radiographic evaluation, the implanted femurs will be fixed into a steel cylinder using a standardized embedding procedure based on a previously reported femur-aligned reference system [38]. During the embedding procedure, a custom alignment fixture will be used, ensuring that the central axis of the femur through fossa piriformis coincides with the central axis of the cylinder. The femurs will be fixed in neutral position on the sagittal plane, using the posterior condylar surface as reference for rotational alignment and at 11^0^ of adduction in frontal plane, to simulate the physiological inclination during single-leg stance [39]. The distal condyles of the intact femur will be embedded into the steel cylinder using the same embedding protocol as described above. The proximal end of each femur will then be prepared for DIC measurements. A layer of matt-white paint, followed by black speckles will be applied creating a high contrast, random speckle pattern.

#### The loading jig

For the purpose of the experimental study, a custom-made mechanical jig has already been designed and manufactured according to the standardized protocol for testing conditions during functional validation of hip prostheses, as reported by Cristofolini and Viceconti [39]. In order to be able to apply the required forces at the desired direction and having the correct magnitude a modular fixture device had to have the following basic characteristics:

- The basic fixture components used for the application of forces on the bone had to be adjustable
- Two load cells had to be used, so that the two forces, on the femoral head and the greater trochanter, could be measured
- Goniometers and rulers are used to verify the correct geometrical features of the fixture

**Fig. 2:**
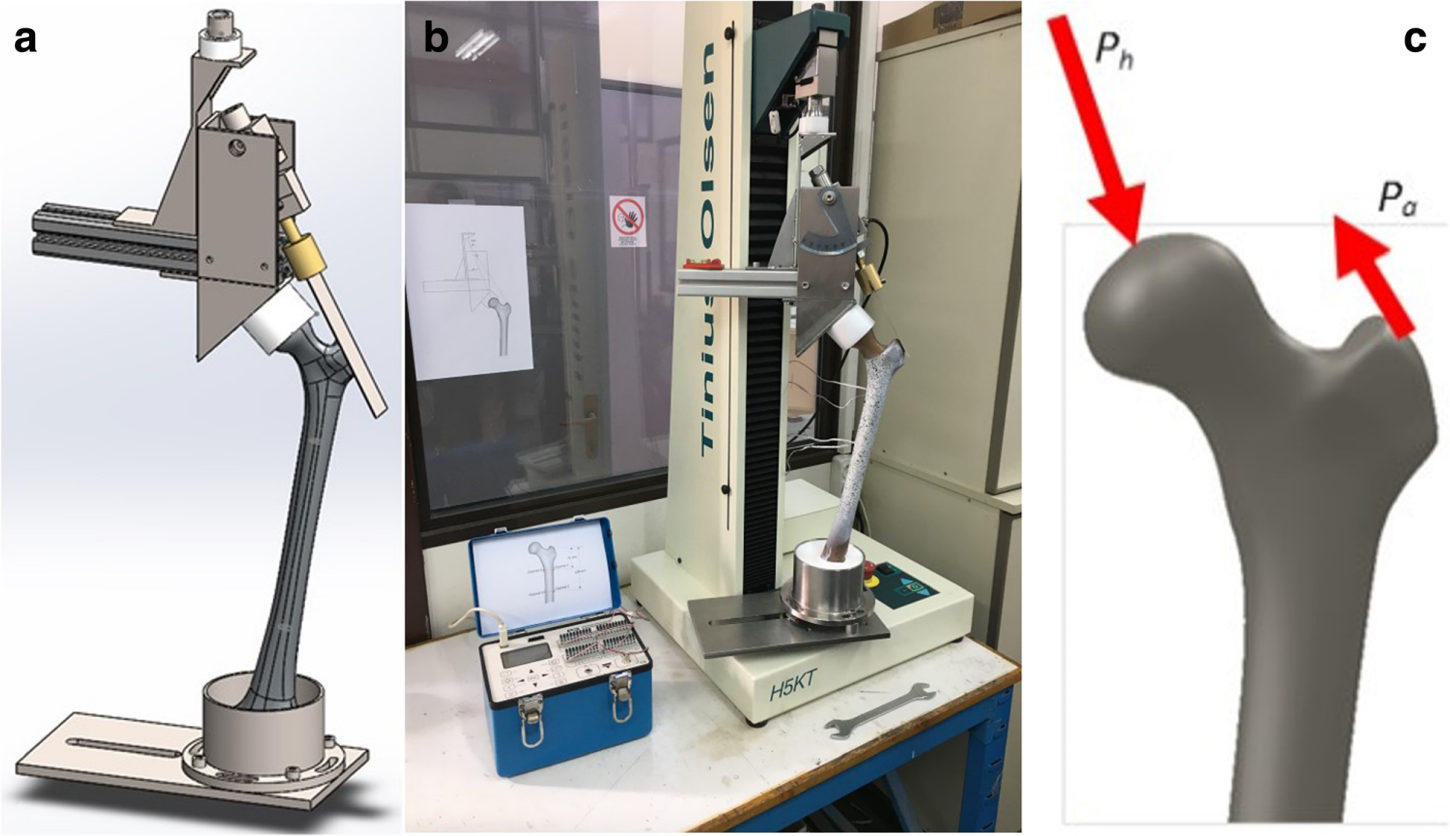
(**a**) Fixture design, (**b**) Establishment for femur experimental biomechanical study and (**c**) Loading system configuration

The design of the developed fixture is presented in Fig. 2a. It comprises two parts; one positioned on the top of the bone to apply forces on the femoral head and the greater trochanter and one used for constraining the femur distally. The top part consists of a metal beam with an acetabular component attached to its undersurface, creating an articulation with the femoral head. The acetabular cup had an inclination of 45^o^ and 0^o^ anteversion. The beam is supported laterally on the femoral head through the acetabular cup and is attached medially to the load cell of a computer-controlled electromechanical testing machine. A system of rulers and goniometers allowed the position and direction of the forces to be accurately controlled.

The test fixture was designed to provide a compressive force to the femoral head through the acetabular cup and a tensile force simulating the contraction of the abductors to the greater trochanter. The phase of gait to be simulated was chosen to be the single-leg stance phase at the moment immediately after heal strike when the highest hip joint load acts [40, 41]. According to previous reports, the abductors exert by far the most relevant load at this phase of gait and thus the other muscles can be neglected in a first approximation [39, 42]. In our experimental fixture, a metallic rod is attached to the lateral aspect of the greater trochanter, using epoxy glue, to simulate the hip abductor muscles. A load cell is used to monitor the force exerted by the abductors in the metallic rod under the different loading conditions. The manufactured jig is presented in Fig. 2b attached to an electromechanical testing machine and applied on an artificial bone. In the same figure the indicator of the load cell measuring the force on the greater trochanter is shown.

#### Loading configuration

A great variability exists in the literature regarding both the magnitude and the direction of forces being included among the different experimental set-ups. In our study, the loading configuration was chosen in accordance with the set-up used by Cristofolini et al. for applying the hip force and the abducting force to the femur [39, 43] (Fig. 2c). According to this loading configuration, a hip joint force (2.47 body weights (BWs) at 29^0^ to the femoral diaphysis) and an abducting force (1.55 BWs, at 40^0^ to the femoral diaphysis) will be applied to the femur.

The non-implanted and implanted femurs will be placed on the testing machine using the custom-made jig. Loads will be applied on the head of the intact composite femur and on the heads of the 28-mm prostheses through the acetabular cup. The specimens will be initially loaded with 100 N and the load will be increased by increments of 100 N up to a total of 1000 N. The strain patterns will be recorded for each loading level.

#### Digital image correlation (DIC)

**Fig. 3:**
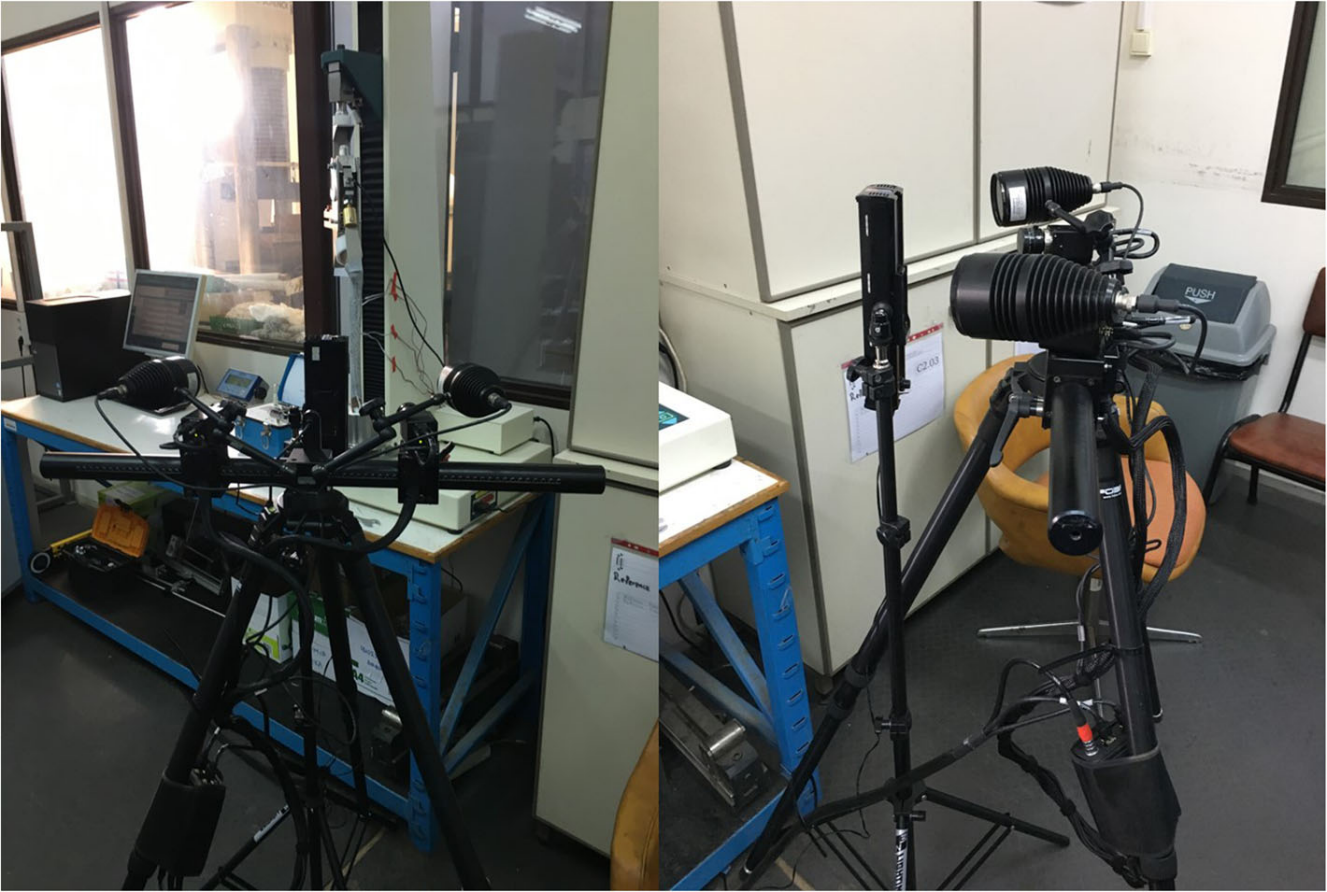
The digital image correlation equipment

The DIC equipment includes two digital cameras that could view the cortex of the femurs from different view angles (Fig. 3). On the femur to be tested a random speckle pattern is created. The non-implanted and implanted femurs will each be placed into the testing machine and load up to 1000 N will be applied according to the configuration described above. The undeformed and deformed speckle patterns on the bone are captured at each loading level. Using the Aramis software the captured imaged are analyzed and detailed 3D contour maps of strain fields are produced for each data set.

#### Statistical analysis

Statistical analysis will be performed using SPSS Statistics (Version 23, IBM SPSS, Armonk, NY, USA). Normal distribution of the parameters of interest, namely axial construct stiffness, failure load, and cycles to failure in each study group will be screened with Shapiro-Wilk test. Homogeneity of variances between the groups will be checked with the Levene test. Significant differences between the two groups will be checked with paired-samples *t* test. A *P* = .05 level of significance will be set for all statistical tests.

### Finite element analysis

**Fig. 4:**
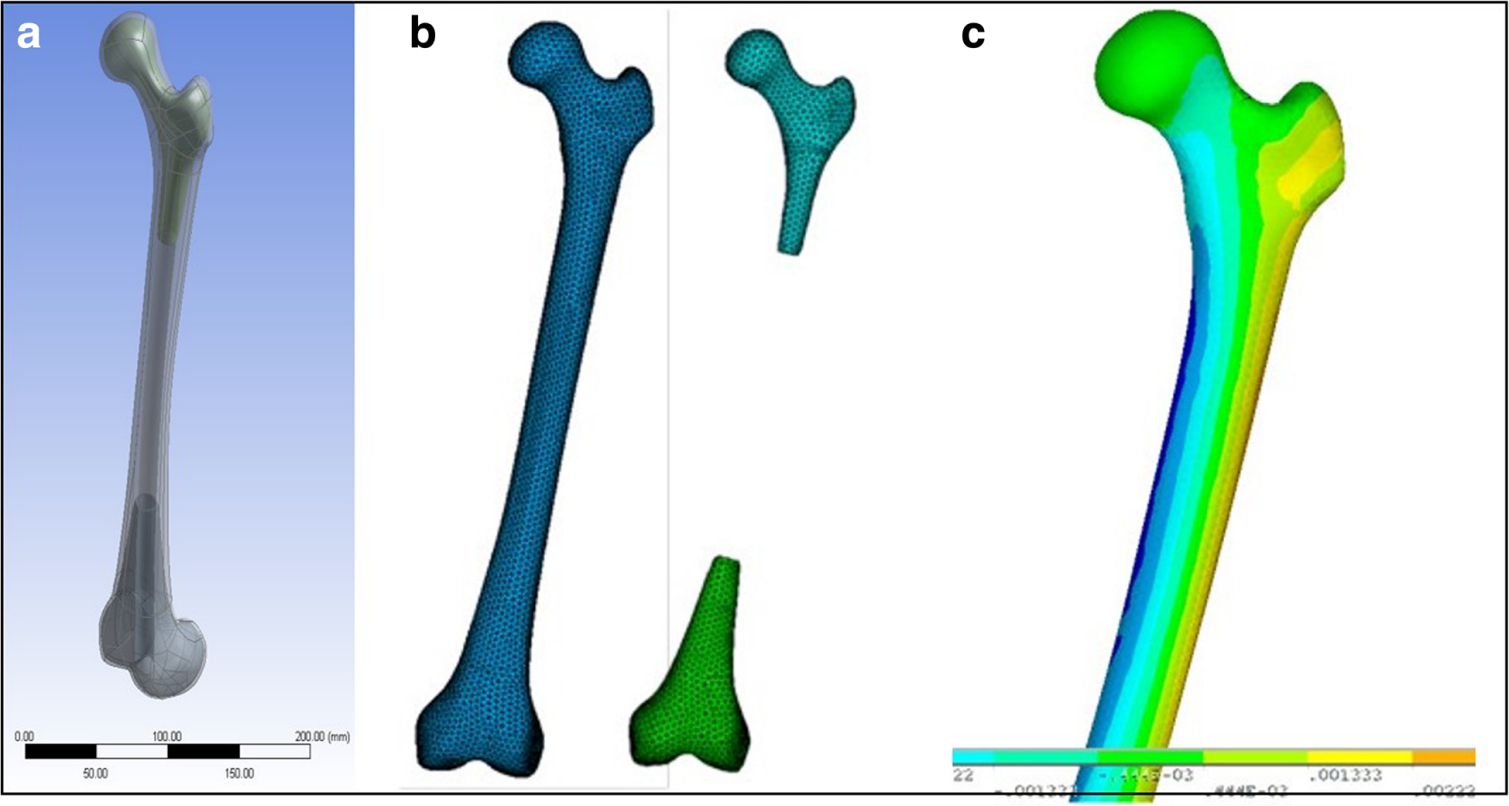
(**a**, **b**) Finite element (FE) models of the cortical and cancellous volumes and (**c**) Indicative strain calculated strain field

A 3D scanner will be used to obtain the geometry of the intact femur and both stems. After modeling the femur, the stems will be placed in the same position as they would be in real surgery. The geometric models of the implanted femurs will be created, based on the orthogonal photographs provided by the cameras during experimental set-up and also the post-implantation antero-posterior and lateral radiographs. Parametric detailed 3D FE models of the bone, the stem and their interface will be developed. Commercially available ANSYS FE code used to develop the femur and implants FE models. For the femur FE model, two different volumes will be meshed to represent the cortical and cancellous material of the bone. Linear tetrahedral elements will be used, as they have been shown to represent smooth surfaces better than cubic hexahedral elements in the femur. Based on a convergence study of the maximum principal strain in FE models with different element sizes (characteristic element lengths: 2 to 10 mm), the optimal element size will be considered. Representative FE models of the cortical and cancellous volumes of the femur are presented in Fig. 4 a, b.

The FE models, would be first validated against mechanical tests on intact bones. The modeled femurs would be used thereafter to calculate strains and stresses at the whole area of the implanted femurs (Fig. 4c) in order to identify highly stressed areas and areas with stress-shielding phenomenon. These results will be compared to the stresses developed during mechanical testing of the two implanted stems and finally stress results will be compared and associated to the results of the clinical study, especially in terms of implant movement or bone absorption.

### Clinical study

#### Study aims

The main purpose of this study is to compare the functional and radiological results of short-stem THA using the TRI-LOCK BPS and the Minima S Femoral Stem, measured by validated clinical scores including gait analysis and standardized radiological parameters up to a minimum of 2 years’ follow-up.

The primary endpoint will be (1) the incidence of all hip-related complications up to 2 years after surgery and (2) change in health-related quality of life assessed with Western Ontario and McMaster Universities Osteoarthritis Index (WOMAC [44]) and the 36-item Health Survey (SF-36) [45] scores up to 2 years. Hip-related complications are defined as intra- and postoperative fractures, dislocation, wound infection, early or late loosening and revision surgery of any implant for any reason. The secondary endpoints at 1- and 2-years’ outcomes include hip function evaluated with the Harris Hip Score (HHS) [46], the Numeric Pain Rating Scale (NPRS), patient satisfaction [47, 48] and the parameters of gait analysis ((a) gait along a 12-m walkway at the patient’s self-selected normal speed, (b) 12-m gait at high speed in respect to patient’s functional status and (c) the Up and Go Test).

#### Design

**Fig. 5:**
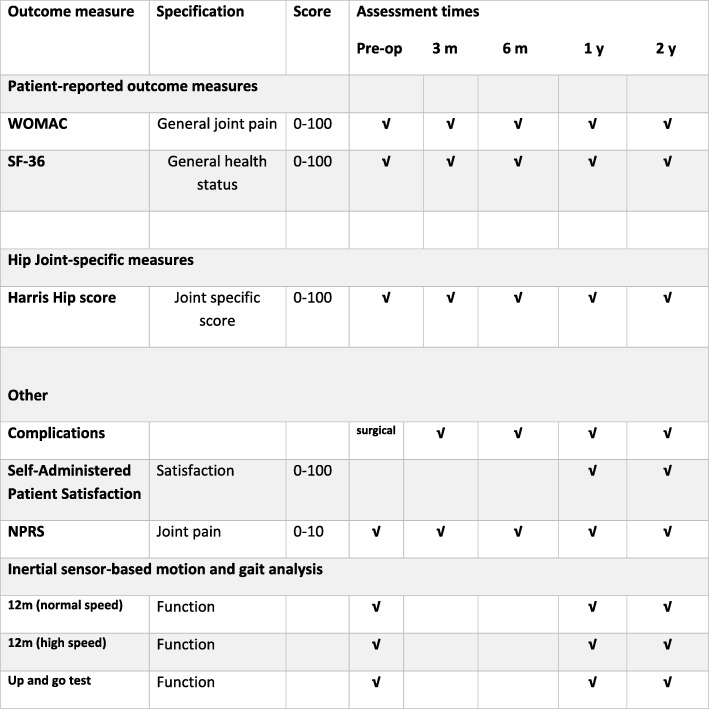
Time schedule and outcome measurements preoperatively and at 3, 6, 12 and 24 months postoperatively

This study uses a prospective, randomized, parallel-group design with blinded treatment and assessment to compare the overall performance of two different short femoral stems, which are theoretically classified in the same main category. This paper is written according to the Standard Protocol Items: Recommendations for Interventional Trials (SPIRIT) 2013 Statement for reporting of clinical trial protocols (Fig. 5, Additional file 1).

The study is sponsored by the University of Patras Research Committee (ELKE) (University of Patras Campus, Rio, Greece). Ethical approval has already been obtained from the Ethics Committee of the University Hospital of Patras (approval number: 36/ 02-03-2016) and the patients’ written consent was obtained prior to participation in the study. The decision was published in the Greek Transparency Portal, called “diavgeia” on 22 April 2016, with a unique Internet Uploading Number that is ADA:6ΝΩ346906Γ-Φ6Ω. The study is registered with International Standard Randomised Controlled Trial Number ISRCTN10096716.

#### Patient selection

Patients will be recruited from the waiting list of the senior surgeon (PM) at the Orthopedic Department of the University Hospital of Patras, Greece. Patients with unilateral hip osteoarthritis for which THA is indicated will be randomized in two different groups. The preoperative raw data will include full demographic profile, patient age, sex, body mass index (BMI), the Charlson Comorbidity Index [49] and a standardized radiological work-up. Eligible patients for short-stem implantation will be between 50 and 80 years old and suffer from (1) primary osteoarthritis, (2) inflammatory arthritis, (3) avascular necrosis and (4) post-traumatic arthritis. Exclusion criteria will include any severe comorbidities affecting functional outcome (i.e., symptomatic lumbar pathology) as well as patients with poor bone stock and any femoral deformity precluding fit and fill in the metaphysis, such in cases of hip dysplasia and severe valgus or metaphyseal deformity secondary to fracture. Participants are free to withdraw from the study at any point or a participant can be withdrawn by the investigator. If withdrawal occurs, it will be documented on a participant “Change of Status” Form. In the event of withdrawal, patients will be invited to provide final primary endpoint data.

#### Randomization procedures

We used stratified block randomization consisting of a random sequence of blocks of 10 consecutive surgical procedures each. Randomization was performed in the operating theater, after anesthetic induction and just before incision, using a sequentially numbered opaque sealed envelope.

#### Intervention

All patients will receive the type of implant to which they have been randomly allocated and thus two groups of patients will be created: group A: Tri-Lock BPS group and group B: Minima S Monolithic Femoral Stem implantation group.

The same senior surgeon (PM) will perform all the arthroplasties with a standardized operative technique through a mini-posterior approach. The femur will be prepared in a broach-only fashion and then the prosthesis will be impacted until a tight metaphyseal fit is achieved. The acetabulum will be prepared in a standardized fashion according to the manufacturer’s manual with the intention of using larger femoral heads (28–32 mm). Ceramic or polyethylene inserts and ceramic or metallic heads in respect will be used according to surgeon’s preference and patients age. All patients in both groups will undergo the same postoperative physiotherapy protocol, which consists of gradual progression from up-to-chair tolerance to ambulation and stair-climbing under the supervision of a certified physical therapist. Patients who manage to complete inpatient physical therapy for independent full weight-bearing would be discharged from further therapy but those with less compliance will continue further rehabilitation.

#### Radiographic evaluation

All antero-posterior pelvis radiographs will be obtained in a similar manner with both legs internally rotated 15^0^, and with bony landmarks (teardrop and lesser trochanter) clearly visible. All radiographic measurements will be performed with AutocadTM software. To test the reliability of the measurements, two independent examiners will review all radiographs. Intra- and inter-rater reliability will be assessed using intra-class correlation coefficients (ICC). Radiographs will be calibrated, and corrected for any magnification based on the known size of the femoral head. Femoral prosthesis fitting, alignment and stability will be assessed. Varus, valgus or neutral implant position will be checked at the first postoperative radiograph by measuring the angulation along the stem relative to the femoral shaft. Stem alignment is considered normal if its deviation from the axis of the femoral shaft is 5^0^ or less. Varus or valgus inclination of the stem relative to the canal of over 5° is defined to be malpositioned [50–52]. In both groups, all follow-up radiographs will be examined for signs of bony ingrowth or signs of loosening and will be classified as osseointegrated, fibrous stable or unstable [53]. To assess stability, length measurements from the superior tip of the greater trochanter to the lateral border of the implant between immediate postoperative and subsequent follow-up visits will be compared. A progressive axial subsidence of > 3 mm, a varus or valgus shift of > 3^0^ and the detection of a complete radiolucent line surrounding the entire porous-coated surface on both the antero-posterior and the lateral radiographs will be considered signs of possible loosening that needs further investigation [50–52, 54–56] such as bone scan. Osteolysis is defined as any discretely localized radiolucency adjacent to the femoral implant, if it is detected on follow-up radiographs but was absent in the immediate postoperative radiographs. The sites of any osteolytic lesions around the femoral component will be recorded according to the classification of Gruen et al. [57]. Given the lack of distal stem in the short femoral components, region from the lower border of the lesser trochanter to the tip of the greater trochanter was defined as zone 1, and the region from the lower border of the lesser trochanter to the femoral neck cut level was defined as zone 7 according to Kim et al. [50]. Pedestal formation will be diagnosed in the presence of bone formation bridging partially or completely the intramedullary canal [6]. Hypertrophies, atrophies, seam formations and spot welds, sclerotic lines in the form of a neocortex as well as periarticular ossifications according to Brooker [58] will be also recorded.

#### Outcome assessment (Fig. 5)

- Patient-reported outcome measures

All eligible patients will complete the Greek version of WOMAC [44] and the Greek version of SF-36 [45] questionnaires as well as the HHS [46] and the Numeric Pain Rating Scale (NPRS).

b. Gait analysis

Supplementary to patient-reported outcome measures, functional assessment will be further investigated by gait analysis, using an inertial measurement unit (IMU: Free4Act [59, 60], Lor An Engineering, Bologna, Italy).

Motion analysis is based on three different physical performance tests: (1) gait along a 12-m walkway at the patient’s self-selected normal speed, (2) 12-m gait at high speed in respect to patient’s functional status and (3) the Up and Go Test. The sensor is composed of a three-axis accelerometer (max range ± 6 g), a three-axis gyroscope (full scale ± 300°/s) and a three-axis magnetometer (full scale ± 6 gauss). The Free4Act sensor is positioned at the dorsal side of the pelvis, centrally between both posterior superior iliac spines. Spatiotemporal gait parameters will be recorded, including: walking speed (m/s), cadence (steps/min) and affected leg step time (s), step length (m), step time irregularity (%), stance and swing phase duration (% of gait cycle). The range of motion (ROM; degrees) of the pelvis in frontal plane (i.e., pelvic obliquity) will be further calculated through the inertial sensor’s inbuilt integration of the gyroscope signals.

c. Reporting of complications/patient satisfaction

All intra-operative and post-operative complications will be recorded. At every outpatient visit, patients will be also asked for the presence or absence of thigh pain. If the answer is “yes,” patients will be further asked whether the pain is at rest or during activity and how often they have thigh pain (all the time, most of the time, some of the time, a little of the time, or never). At the 1-year appointment and yearly thereafter, patient satisfaction [47, 48] with the outcome will be assessed and categorized as: overall satisfaction, satisfaction with pain relief, functional improvement to perform daily activities and satisfaction with ability to perform recreational activities. Patients will be classified as very satisfied, somewhat satisfied, somewhat dissatisfied and dissatisfied.

#### Follow-up assessments

Clinical and radiographic follow-up assessments are scheduled at 3, 6 and 12 months and annually thereafter for a minimum of 2 years’ follow-up. Each visit will include clinical and radiological examination with antero-posterior and frog-leg lateral views of the pelvis. All patients would again fill up the HHS, WOMAC, SF-36 and NPRS scale. At the 1-year appointment and yearly thereafter, patient satisfaction with the results of THA will be assessed and gait analysis will be also performed (Fig. 5).

#### Analysis

##### Power and sample size calculation

The minimal clinically important differences (MCID) for sample calculation have been defined according to the literature at 25 points for the WOMAC score, 20 points for the HHS and 12% difference for the SF-36 score. In order to achieve a 80% power or better the effect size is expected to lie between 0.25 and 0.6, suggesting a sample size of 45 patients in each group [61].

##### Statistical analysis

Analysis will be by intention to treat. Differences between groups will be estimated using either the *t* test or Wilcoxon test at the 5% significance level to compare the distribution of the primary endpoint between the treatment samples. The patient outcome scores will be assessed with repeated measures analysis of variance (ANOVA) models to account for the repeated data collection time points. Results will be presented as an adjusted mean difference with its corresponding 95% confidence intervals.

## Discussion

As THA is increasingly being performed in patients who are younger and more active, the need for better implant design for bone and soft tissue preservation is mandatory; short, uncemented femoral implants are an alternative considering that they provide the following theoretical advantages [62–68]: (1) demonstrate more physiological load transfer distribution to the proximal femur, reducing proximal stress shielding, (2) facilitate minimally invasive surgical techniques, (3) preserve metaphyseal bone stock by a more proximal fixation, (4) provide more favorable conditions in the potential revision setting, (5) are viable alternatives in cases of metaphyseal-diaphyseal mismatch (e.g., excessively bowed femurs, deformed bones, narrow Dorr Type A diaphysis) and (6) have decreased rates of thigh pain.

The fact is that all the above benefits still need to be sufficiently validated and also short stems should provide better or, at least equal outcomes compared to those already have been reported for standard stems in long-term clinical studies. One major problem is that there are several variations and design philosophies of short stems in the market and their types and categories are sparse and unclassified [69, 70]. Recently, Gómez-García et al. [71] published a detailed classification of short stems by means of a nomenclature that describes them accurately, taking into consideration several variables, such as (1) the anatomical region that they occupy or invade, (2) basic geometric design, (3) main stress distribution zones, (4) bone resection level and (5) the orientation axes used for insertion. According to this classification, which we have adopted, the Tri-Lock BPS and Minima S stems are classified as short stems type C, which means that they occupy the cervico-metaphyseal-diaphyseal area. The Tri-Lock BPS stem is further classified as a straight, wedged stem with major stress transmission areas, the calcar and the diaphysis and a rectangular cross-section and the Minima S stem as a curved, wedged stem with calcar and proximal diaphyseal loading distribution and an oval-trapezoidal cross-section.

In our study, we aim to conduct a comprehensive assessment, by means of a comparative clinical study as well as biomechanical testing and FEA of two different short femoral stems, which are theoretically classified in the same main category. For this reason, we decided to compare a newly introduced short stem, Minima S, for which clinical performance data are lacking with an older generation short stem, Trilock BPS, with an established performance record in short to midterm follow-up [72–74]. We can hypothesize that even these subtle variations in geometric design between these two stems, which are classified within the same subgroup, may create different loading characteristics and thus dissimilar biomechanical behaviors, which in turn could have an influence to their clinical performance. Consequently, if this scenario is finally confirmed, the conclusions of the present study could not be extrapolated to all short stems even if they belong to the same category.

Today’s demographic profile and demands of patients for hip replacement surgery emphasizes the importance of using more accurate assessment tools that can detect changes in functional ability. Despite the fact that patient-reported outcomes measures (PROMs) are widely used in the literature to assess outcome following total hip arthroplasty, they represent only a self-reported perception of a patient’ s functional status and are subject to patient reporting bias. Furthermore, several PROMs suffer from a ceiling effect [75], which means that a substantial proportion of participants reach the maximum score at the same period and thus the extent of a possible further improvement may not be determined. Hence, in our study we decided to include an objective tool supplementary to PROMs for the assessment of functional ability after THA, that is the gait analysis using an inertial sensor. Despite the fact that stereophotogrammetric systems are considered the gold standard for clinical gait analysis, they require a specially equipped laboratory and they are time-consuming and expensive. More recently, inertial sensors have developed as reliable tools alternative to stereophotogrammetric systems and have also demonstrated responsiveness to postoperative changes in patients with hip osteoarthritis [60, 76].

### Limitations

#### Clinical study

One important limitation is the relatively short follow-up period of 2 years. Only long-term results should be considered valid, but as other studies have previously reported, the most important issues in achieving long-term fixation seems to be the initial fitting and the prevention of early progressive stem migration [77–80].

Second, we use computed radiological methods that lack the quantitative accuracy of radiostereometric analysis (RSA) [81, 82] and dual-energy x-ray analysis (DEXA) [83] in regards to measurement of subsidence, remodeling, femoral bone loss and stress-shielding effect. Our qualitative assessment of bone remodeling and stress-shielding effect will be made from plain radiographs. Engh et al. [84] reported a successful method of measuring bone remodeling on radiographs and confirmed the radiographic results with histologic examination.

Third, in our study, all operations will be performed by a single surgeon and thus the reported results could potentially reflect one surgeon’s experience. Nevertheless, the technique for implanting metaphyseal fitting short-stem prostheses has many similarities to that for inserting stems of conventional length and thus the technique and outcomes can be expected to be replicable.

#### Biomechanical study

In our work, we decided to use composite femoral bones instead of cadaveric human specimens. Composite femoral bones have been independently tested and proven to demonstrate a biomechanical behavior similar to human cadaveric specimens [37] but can increase the sensitivity of the study, as they exhibit low interspecimen variability regarding bone geometry and mechanical properties between the specimens. On the other hand, cadaveric specimens pose other problems, such as availability and the requirement of specific storage conditions and preparation techniques, that can significantly affect their mechanical properties.

The DIC technique [24, 26, 85–88] a relatively new concept in the field of biomechanics for strain measurement, has been adopted in our study like an alternative approach to the older technique of strain gauges as it provides the advantages of reduced specimen preparation time and full-field data analysis. Despite their widespread application, strain gauges require detailed surface preparation and provide strain results only at the application site.

Finally, our test set-up will not include all of the surrounding soft tissues, due to the inherent difficulty of such a simulation in the laboratory setting. Nevertheless, in accordance with previous reports [39, 42] abductor muscle forces have been demonstrated to exert the greatest effect on strain patterns in the proximal aspect of the femur and thus the other muscles can be neglected in a first approximation. Recent experimental studies evaluating femoral prostheses have shown a tendency towards being as simple as possible, including only the abductor muscles [27, 89, 90]. We are also aware that cortical strain measurement cannot directly be compared with in vivo performance because composite bone models fail to represent a vital bone with blood supply, which plays a role in bone adaptation. Thus, our test set-up describes a scenario of the first postoperative period, disregarding any process of osseous integration. However, these testing conditions could provide useful data on the mechanical behavior of the implants, which in turn may reflect the bone adaptation process. Concerning FE models, the usual limitations are present. The bone is modeled in a simplified way without accounting for factors, such as the bone quality, which influence the decision-making in real surgery. Moreover, isotropy of bone remains an assumption whereas its actual behavior is close to an orthotropic material. Our study has been designed to compare the biomechanical properties, based on both biomechanical tests and FE models. Viceconti et al. [91] and Cristofolini et al. [92] recommend the combination of the experimental and numerical methods, each having its own limitations and advantages, as this seems to have synergistically effects. Biomechanical studies evaluating acute changes in the strain patterns in femoral bones after insertion of femoral prostheses are valuable in the assessment of the impact of different implant variables on the load transfer to the bone. Nevertheless, we are aware that neither experimental studies nor numerical analyses can be used uncritically to predict the clinical performance of different prostheses. In order to overcome these limitations regarding clinical relevance, we decided to conduct a simultaneous prospective clinical study, where the remodeling process around short-stem prostheses could be closely monitored, by means of a detailed radiological method.

## Additional file

Additional file 1: Standard Protocol Items: Recommendations for Interventional Trials (SPIRIT) 2013 Checklist: recommended items to address in a clinical trial protocol and related documents*. (DOC 127 kb)

## Data Availability

The data sets used and analyzed during the current study are available from the corresponding author on reasonable request.

## Abbreviations

3D: Three-dimensional
BMI: Body mass index
BPS: Bone Preservation Stem
BWs: Body weights
DEXA: Dual-energy x-ray analysis
DIC: Digital image correlation
FE: Finite element
FEA: Finite element analysis
HHS: Harris Hip Score
ICC: Intra-class correlation coefficients
IMU: Inertial measurement unit
MCID: Minimal clinically important differences
NPRS: Numeric Pain Rating Scale
PROMs: Patient-reported outcomes measures
ROM: Range of motion
RSA: Radiostereometric analysis
SPIRIT: Standard Protocol Items Recommendations for Interventional Trials
THA: Total hip arthroplasty
WOMAC: Western Ontario and McMaster Universities Osteoarthritis Index
